# Radiological Configuration of the Vestibular Aqueduct Predicts Bilateral Progression in Meniere's Disease

**DOI:** 10.3389/fneur.2021.674170

**Published:** 2021-06-08

**Authors:** David Bächinger, Bernhard Schuknecht, Julia Dlugaiczyk, Andreas H. Eckhard

**Affiliations:** ^1^Department of Otorhinolaryngology, Head and Neck Surgery, University Hospital Zurich, Zurich, Switzerland; ^2^University of Zurich, Zurich, Switzerland; ^3^Medical Radiological Institute MRI, Zurich, Switzerland

**Keywords:** endolymphatic hydrops, endolymphatic sac, vestibular aqueduct, prognosis, MRI, CT, imaging

## Abstract

**Objective:** Meniere's disease (MD) progresses from unilateral to bilateral disease in up to 50% of patients, often chronically and severely impairing balance and hearing functions. According to previous studies, 91% of bilateral MD patients demonstrate bilateral hypoplasia of the endolymphatic sac (ES) upon histological and radiological examination of their inner ears. Here, we seek to validate a radiological marker for ES hypoplasia that predicts the risk for future progression to bilateral MD in individual patients.

**Methods:** Patients with unilateral MD and radiological evidence for ES hypoplasia in either the clinically affected inner ear (cohort MD_uni_-hp_uni_) or both inner ears (cohort MD_uni_-hp_bi_) were included. Given our hypothesis that ES hypoplasia critically predisposes the inner ear to MD, we expected progression to bilateral MD only in the MD_uni_-hp_bi_ cohort. To investigate eventual progression to bilateral MD, clinical, audiometric, and imaging data were retrospectively collected over follow-up periods of up to 31 years.

**Results:** A total of 44 patients were included in the MD-hp_uni_ (*n* = 15) and MD_uni_-hp_bi_ (*n* = 29) cohorts. In line with our radiology-based predictions, none (0/15) of the MD-hp_uni_ patients exhibited progression to bilateral MD, whereas 20/29 (69%) MD-hp_bi_ patients have already progressed to bilateral MD. Using the Kaplan–Meier estimator, bilateral disease progression would be observed in 100% of MD-hp_bi_ patients 31 years after the initial diagnosis with an estimated median time to bilateral progression of 12 years. The nine MD-hp_bi_ patients who, so far, remained with unilateral disease demonstrated a median time since initial (unilateral) MD diagnosis of only 6 years and are thus still expected to progress to bilateral disease.

**Conclusion:** Progression to bilateral MD adheres to predictions based on the radiological presence or absence of ES hypoplasia. This prognostic tool, if validated by prospective long-term studies, will provide clinically relevant information about a patient's future disease burden and will help to select more personalized treatment regimens.

## Introduction

Meniere's disease (MD), a chronic inner ear disorder, causes fluctuating vestibular and auditory symptoms and exhibits a highly variable disease course among patients (1, 2). Most severely affected are the 10–50% of patients (3–5) in whom the disease progresses to bilateral MD, often years to decades after its initial (unilateral) manifestation. Due to bilateral vestibulopathy, patients with bilateral MD experience chronic debilitating vestibular symptoms, such as oscillopsia and imbalance, and a broad range of cognitive and emotional impairment (6). Moreover, bilateral MD often leads to severe to profound hearing loss with unserviceable speech discrimination (7, 8). A biomarker for future bilateral disease would allow clinicians to identify those patients, to preemptively counsel them about the expected disease course, and to personalize therapy regimens in clinically meaningful ways.

In this study, radiological evidence for endolymphatic sac (ES) hypoplasia (9, 10), i.e., the suspected etiopathology in approximately 30% of MD patients, with the designated endotype “MD-hp” (11), was used to prognosticate disease laterality. In a recent human temporal bone study, either of two pathologies of the ES, i.e., degeneration or developmental hypoplasia, was consistently found in cases with clinical MD (9). These ES pathologies (“endotypes”) can be linked to the pathogenesis of endolymphatic hydrops, a histopathologic and radiologic marker of MD (9, 12). The subtype of ES pathology correlates with the course of the vestibular aqueduct, which–in contrast to the ES epithelium–can be visualized in clinical imaging (10). In MD, the course of the vestibular aqueduct can therefore be used as a radiologic surrogate marker for the underlying subtype of cellular ES pathology (10). Using this marker, it has been shown in clinical patients that the pathologic endotypes are associated with differing clinical phenotypes (11). In MD-hp patients, one or both inner ears may exhibit ES hypoplasia. However, to date, it is not clear whether ES hypoplasia critically predisposes to MD. Here, we hypothesized that progression to bilateral MD only occurs in patients with bilateral ES hypoplasia (cohort MD_uni_-hp_bi_) but not in those with unilateral ES hypoplasia (cohort MD_uni_-hp_uni_).

## Materials and Methods

### Ethics Approval

This study was approved by the local ethics committee (KEK-ZH-Nr. 2016-01619/2019-01006) in accordance with the Declaration of Helsinki and its amendments. Informed consent has been obtained from all participants.

### Study Design and Participants

Patients from an interdisciplinary tertiary neurotology center fulfilling the following inclusion criteria were included into the study between August 2019 and December 2020: (i) patients with an initial diagnosis of unilateral definite MD (1, 13), (ii) radiological evidence for uni- or bilateral ES hypoplasia (endotype MD-hp), (iii) endolymphatic hydrops in the affected ear (see next paragraph), and (iv) age ≥18 years. The time point of first MD manifestation was defined by the first reported episode of spontaneous vertigo lasting >20 min or by the first audiometrically documented hearing loss, which matched the MD diagnostic criteria (1, 13). Patients with secondary Meniere's syndrome due to a known pathology were excluded (14, 15). Bilateral progression was defined as recurrence of vertigo attack(s) and audiometrically documented hearing loss in the second ear (16).

### Temporal Bone Imaging

At the time of initial clinical work-up, 3 Tesla gadolinium-enhanced MRI (Gd-MRI) of the temporal bones was performed using a 32-channel phased array coil to visualize endolymphatic hydrops (17, 18) and to exclude other intra- or retrolabyrinthine pathology. Endolymphatic hydrops grading was performed separately for the cochlea and the vestibule (18) by an experienced neuroradiologist (BS). In some patients, additional high-resolution CT (HRCT) of the temporal bones was performed, e.g., to exclude a dehiscence syndrome.

### Vestibular Aqueduct Measurements and Patient Endotyping

**Figure 1:**
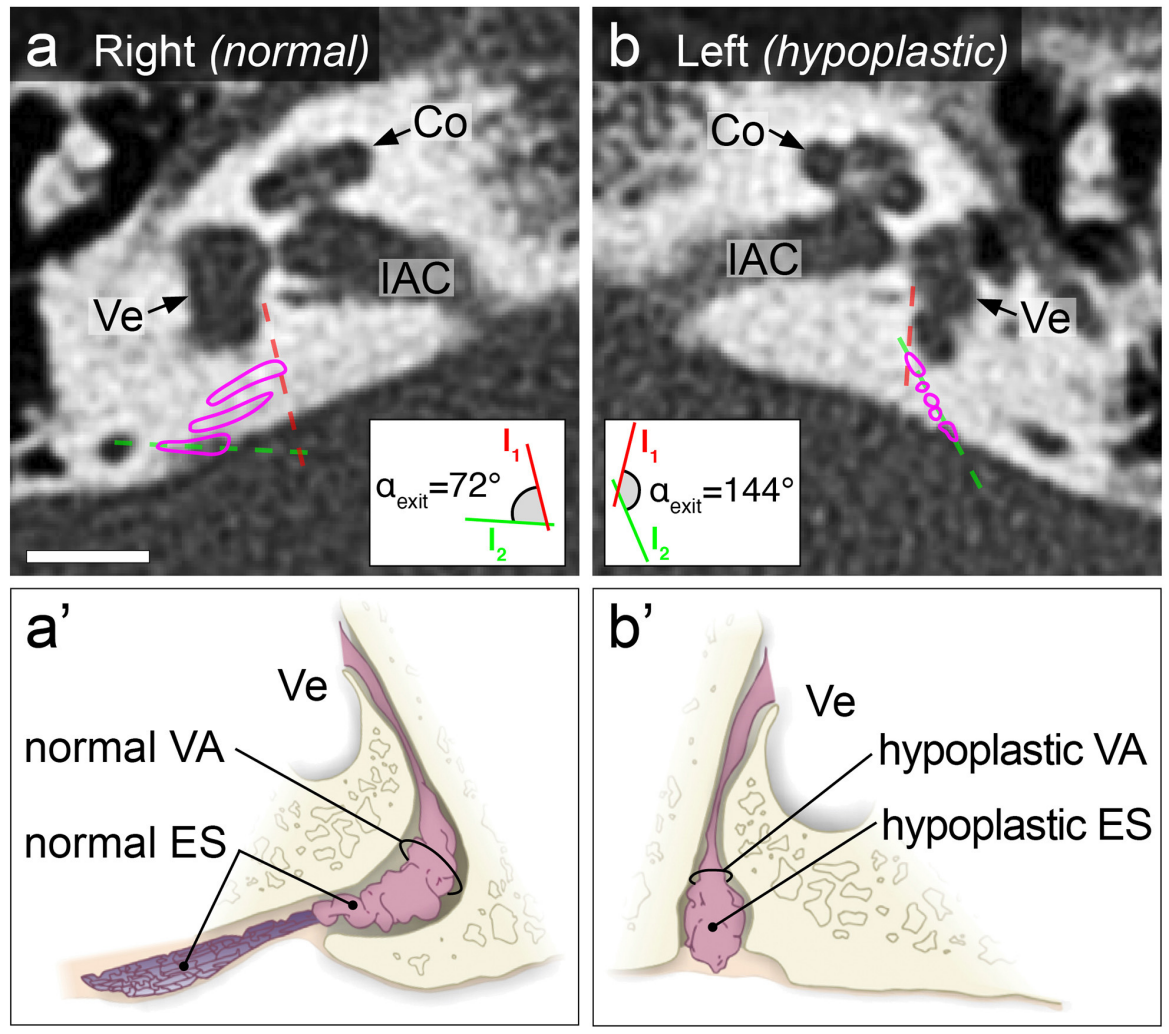
Illustration of normal and hypoplastic ES morphology in temporal bone HRCT from an MD_uni_-hp_uni_ patient. Axial plane CT images show a normal, bent course of the 2D-reconstructed vestibular aqueduct on the right (magenta outlines in **(a)**; ATVA with α_exit_ = 72°), indicating the presence of a normal ES. The panel below shows a drawing of a normal ES located within a normal vestibular aqueduct **(a′)**. On the left side, an abnormal, straight course of the vestibular aqueduct was found (magenta outlines in **(b)**; ATVA with α_exit_ = 144°), indicating a hypoplastic ES. The panel below shows a drawing of a hypoplastic ES located within a hypoplastic vestibular aqueduct **(b′)**. This patient had a 9-year history of left-sided MD. Insets show the geometric measurement results for the ATVA, according to previously described methods (10). Co, cochlea; ES, endolymphatic sac; IAC, internal auditory canal; VA, vestibular aqueduct; Ve, vestibule. Scale bar: 5 mm. **(a′,b′)** are adapted from Eckhard et al. (9), under the terms of the Creative Commons CC BY license (http://creativecommons.org/licenses/by/4.0/).

The angular trajectory of the vestibular aqueduct (ATVA) was determined for each inner ear in HRCT data if available or in Gd-MR imaging data (3D real inversion recovery sequence). An ATVA with an angle α_exit_ >140° indicated ES hypoplasia, and an α_exit_ <120° indicated a normal ES (Figure 1), as defined previously (9, 10). Patients with α_exit_ >140° and <120° on the clinically affected and the non-affected side, respectively, were assigned to the MD_uni_-hp_uni_ cohort, whereas those with an α_exit_ >140° on both sides were assigned to the MD_uni_-hp_bi_ cohort.

### Statistical Analysis

Values are reported as absolute numbers (percentage) or means with standard deviation (SD) and range. Continuous variables were analyzed using a two-tailed Student's *t*-test for independent samples. For binary variables, a Fisher's exact test was performed. A *p* < 0.05 was considered as statistically significant. The Kaplan–Meier method was used to quantify the percentage of patients with progression to bilateral MD. The Log-rank (Mantel–Cox) test was used to compare Kaplan–Meier curves. Follow-up times were censored for all cases at the time when the final analysis was initiated (January 2021). Statistical analyses were performed using Prism for Apple Macintosh, version 7.0 (GraphPad Software, Inc., La Jolla, CA, USA).

## Results

### Clinical Features of the Study Cohorts

A total of 44 patients were included between August 2019 and January 2020. From those, 15 patients were assigned to the MD-hp_uni_ cohort, and 29 patients to the MD-hp_bi_ cohort based on the radiological criteria defined above (Table 1). In 35/44 (80%) patients, HRCT was available and used for vestibular aqueduct measurements. In the remaining 9/44 (20%) patients, Gd-MRI was used for vestibular aqueduct measurements. Male to female ratio, mean age at MD onset (time point of initial diagnosis), and range of disease duration did not significantly differ between both cohorts (Table 1). Among the 44 MD-hp patients, 14 (32%) had a positive family history for MD, 3 (7%) had migraine, and none had a known autoimmune disorder.

**Table 1:** Demographics and clinical characteristics of the MD-hp cohorts.

	**MD-hp (*n =* 44)**	**MD-hp_**uni**_ (*n =* 15)**	**MD-hp_**bi**_ (*n =* 29)**	**MD-hp_**uni**_ vs. MD-hp_**bi**_**
Male to female ratio, no. (%)	37:7	13 (87%):2 (13%)	24 (83%):5 (17%)	*p* = 0.99
Mean age at onset, years (SD)	39.0 (11.6)	39.1 (7.8)	39.0 (13.2)	*p* = 0.96
Mean disease duration, years (SD, range)	14.7 (12.1)	10.7 (7.7, 2–30)	16.8 (13.7, 1–31)	*p* = 0.12
Unilateral to bilateral MD ratio, no. (%)	24 (55%):20 (45%)	15 (100%):0 (0%)	9 (31%):20 (69%)	*p* < 0.0001

### Progression to Bilateral MD Is Exclusively Observed in the MD-hp_bi_ Cohort

**Figure 2:**
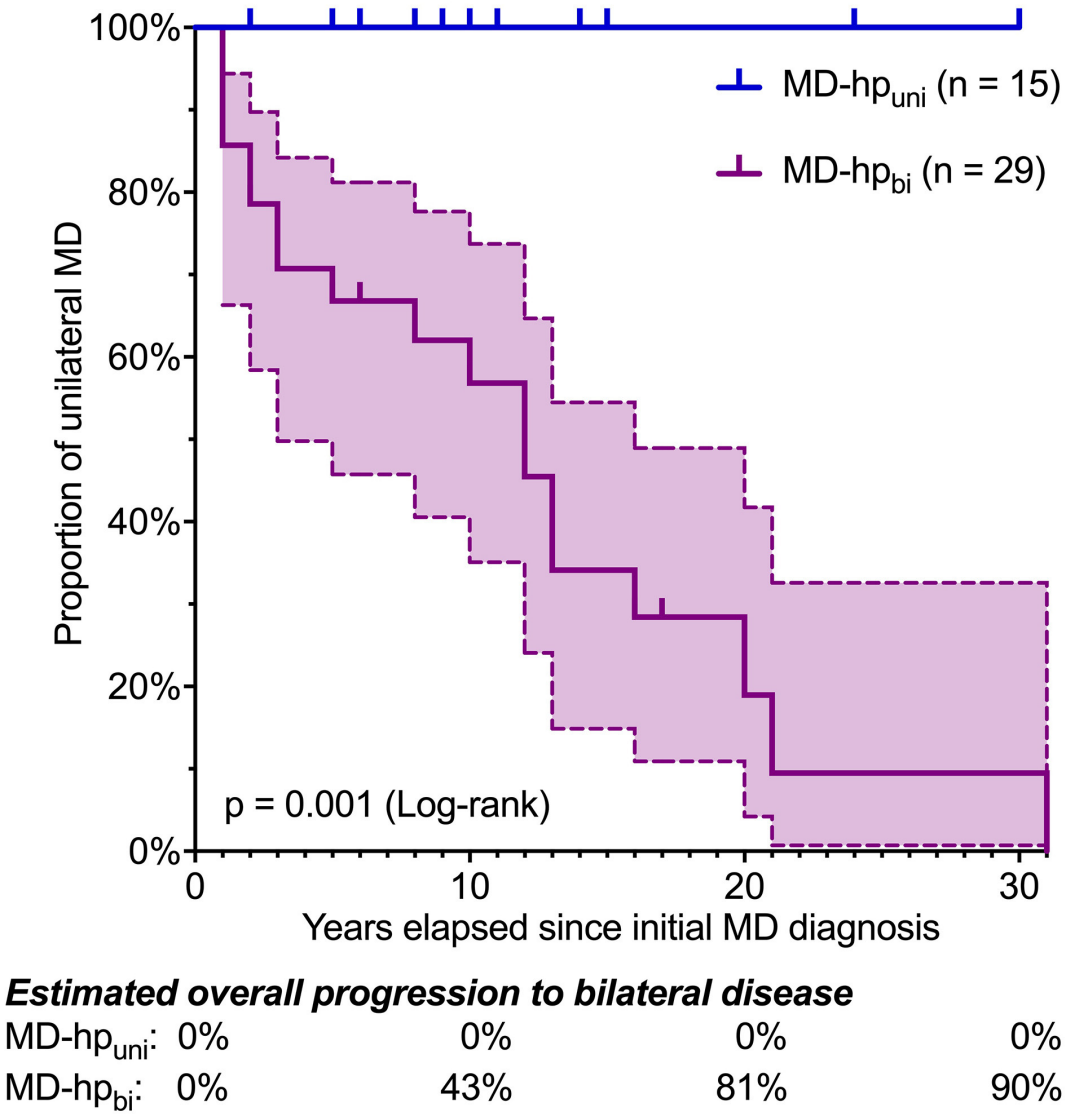
Kaplan–Meier plot for progression to bilateral MD in the MD-hp_uni_ and MD-hp_bi_ cohorts. Ticks indicate censored cases. In the MD-hp_bi_ cohort, median time to bilateral progression was 12 years. None of the MD-hp_uni_ patients developed bilateral disease within 30 years. Dashed lines indicate 95% confidence intervals.

Within 30 and 31 years from onset of MD, none (0/15) of the MD-hp_uni_ patients and 20/29 (69%) MD-hp_bi_ patients progressed to bilateral MD, respectively (*p* = 0.001; Figure 2, Table 1). In MD-hp_bi_ patients, the estimated median time to bilateral progression was 12 years. The proportion of patients with bilateral MD was estimated to increase to 43% after 10 years and to 90% after 30 years with unilateral MD, respectively (Figure 2).

### Prospective Observation of Bilateral Disease Progression in an MD_uni_-hp_bi_ Patient

**Figure 3:**
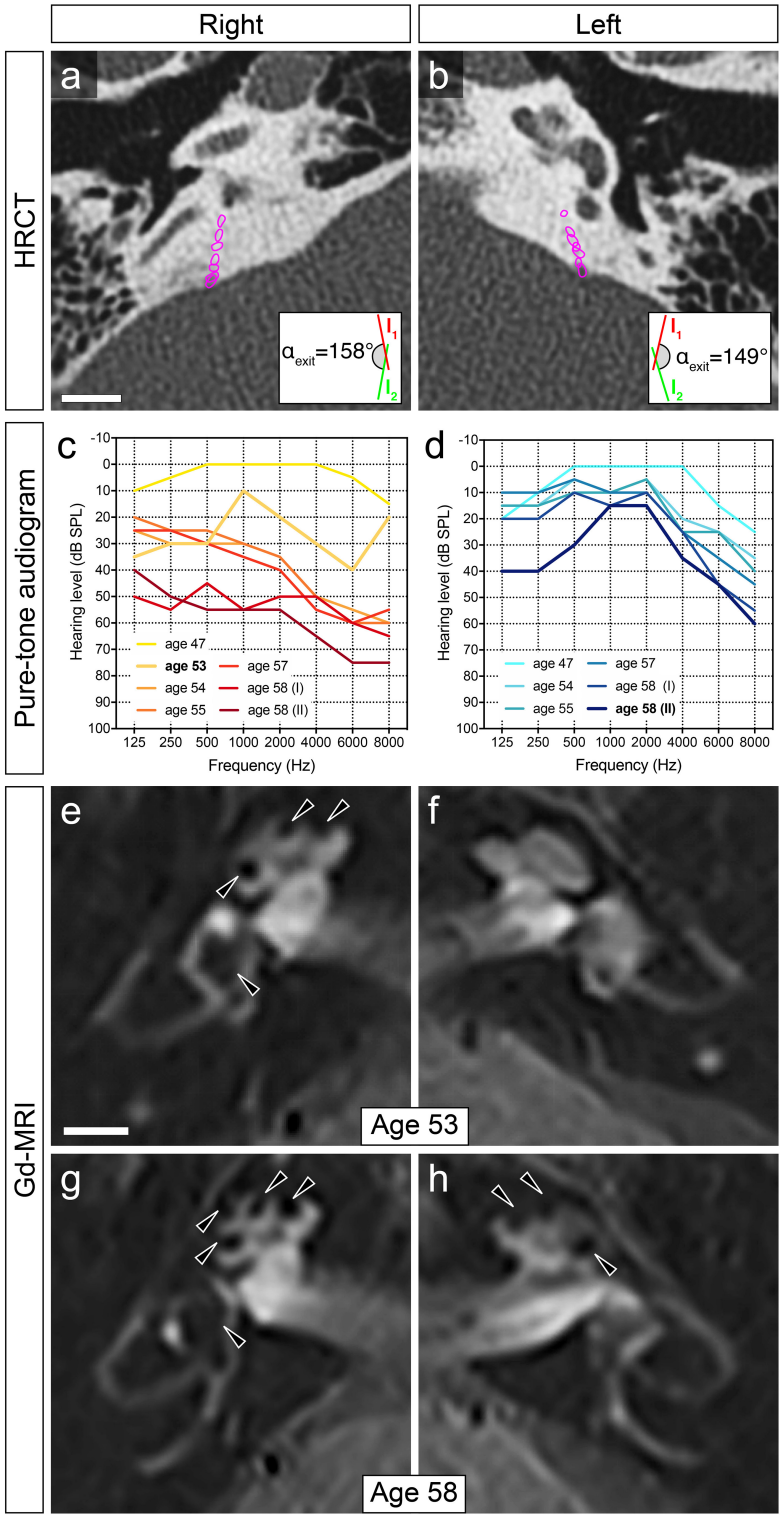
Exemplary clinical case progressing to bilateral disease. **(a,b)** In this patient, ATVA measurements on temporal bone HRCT images performed at age 53 determined angles (α_exit_) consistent with bilateral ES hypoplasia (2D-reconstructed course of the vestibular aqueduct outlined in magenta). Scale bar: 5 mm. **(c,d)** Audiogram data (air conduction thresholds) before MD first manifested (age 47), after onset of unilateral (right) MD [ages 53–58 (I)], and shortly after progression to bilateral disease [age 58 (II)]. **(e,f)** Gd-MRI at the time point of initial MD diagnosis (age 53) demonstrated grade 1 (18) cochleovestibular hydrops (black arrowheads) in the right inner ear. **(g,h)** Sequential Gd-MRI, after clinical and audiometric data indicated progression to bilateral MD (age 58), demonstrated grade 1 cochleovestibular hydrops in the right and left inner ears. Scale bar: 5 mm.

This male patient, who was followed up in our neurotology center between age 53 and 58, was prognosticated to develop bilateral MD based on temporal bone HRCT imaging signs for bilateral ES hypoplasia (Figures 3a,b). The patient initially was seen at age 53 with complaints of monthly, spontaneous vertigo episodes (up to 6 h), accompanied by hearing loss and fullness in his right ear. Pure tone audiometry at the time showed moderate sensorineural hearing loss at low and high frequencies (“peak pattern”) on the right side (Figures 3c,d). Inner ear Gd-MRI demonstrated grade 1 cochleovestibular hydrops in the right ear (Figures 3e,f), supporting the clinical diagnosis of right definite MD. The patient was started on betahistine (48 mg twice daily). Over the following 5 years, in which he was followed up at 6–12-month intervals, he reported only one more vertigo attack. During this time, right-sided hearing progressively deteriorated to a moderate sensorineural hearing loss that affected all frequencies [Figure 3c, age 53–58 (I)]. A left-sided age-appropriate high-frequency sensorineural hearing loss was observed during that time [Figure 3d, age 54–58 (I)]. At age 58, the patient again experienced weekly, hours-long spontaneous vertigo episodes as well as a new hearing loss in his previously unaffected left ear. Pure tone audiometry at that time demonstrated a new low-frequency hearing loss in the left ear (Figure 3d), and sequential Gd-MRI of the inner ears showed a new cochlear hydrops grade 1 in the left ear (Figures 3g,h). The clinical diagnosis was revised accordingly to bilateral definite MD. Of note, a further progression of high-, but not low-frequency sensorineural hearing loss was observed for the right ear supporting the notion of an anticipated age-related decline in the right ear, whereas vertigo attacks now originated from the left ear [Figures 3c,d, age 58 (I) and 58 (II)].

## Discussion

MD-hp patients statistically have a 25% risk for developing bilateral disease, which is 5-fold higher than other MD patients (11). Thus, providing MD-hp patients with a personalized prognosis, instead of a mere statistical one-in-four chance for bilateral disease progression, would advance the clinical management of this patient group, which is at highest risk for a severe disease course, in a clinically relevant manner.

MD-hp patients are distinguished by a developmentally rudimentary, i.e., hypoplastic, ES (Figure 1b′), which has been initially described in sporadic MD cases (19–22) and was more recently demonstrated as a consistent finding in about 30% of pathology cases with a clinical MD diagnosis (9)—now designated as the MD-hp patient group (11). Pathophysiologically, the absence of normal ion transport mechanisms in the hypoplastic ES epithelium is believed to be the key pathology that causes disturbances of the inner ear fluids and, ultimately, endolymphatic hydrops and clinical symptoms (9, 23). ES hypoplasia is consistently associated with a hypoplastic bony VA, which can be radiologically visualized and was established as a clinical surrogate marker (ATVA) for the presence of ES hypoplasia (10).

This present preliminary and previous (11) data suggest that the radiological ATVA marker has absolute positive and negative predictive power for making this personalized prognosis. If ultimately validated by long-term prospective studies, this would allow clinicians (i) to counsel patients about aspects of their future disease course, in particular about the long-term impacts on hearing and balance functions; (ii) to make better decisions on whether ablative therapies, such as intratympanic gentamicin or vestibular neurectomy, can be considered; and potentially also (iii) to screen family members and children from MD-hp patients for their risk of developing uni- or bilateral MD. Furthermore, the ATVA marker enables to define clinically more homogenous subgroups of MD patients for future (genomic) studies.

In bilateral MD, non-ablative treatments, such as intratympanic steroids, should be preferred over ablative treatments, which should be avoided (16). Using the ATVA marker, progression to bilaterality can be anticipated in MD-hp_bi_ patients and virtually excluded in MD-hp_uni_ patients. Therefore, this clinical tool may allow to obviate iatrogenic bilateral vestibular loss and/or hearing loss due to ablative treatments in MD-hp_bi_ patients. On the other hand, in MD-hp_uni_ patients with an infinitesimal risk for bilateral progression, ablative treatments, such as intratympanic gentamicin administration, may be liberally used.

ES surgery is commonly considered as a non-ablative treatment of MD (16). It should be noted, however, that in MD-hp patients, the ES is hypoplastic and anteriorly displaced. These anatomical abnormalities impede the surgical approach of the ES *via* the commonly used transmastoid route; furthermore, the hypoplastic ES occasionally does not exit the temporal bone at all. Of note, the surgical non-visualization rate of the ES during ES surgery corresponds to the percentage of MD-hp patients among all MD patients (11, 24). Although there is lacking evidence for a beneficial effect of ES surgery (25), this “non-ablative” surgical treatment–if considered–may therefore be particularly avoided in MD-hp patients.

Notably, among other proposed markers for predicting bilateral MD (2, 18), cervical vestibular-evoked myogenic potential (cVEMP) metrics were retrospectively found to be altered, with high specificity and sensitivity, in clinically yet unaffected contralateral ears 24–288 months before clinical symptoms manifested (26, 27). The actual time course of these cVEMP changes and their earliest appearance during the presymptomatic phase remain to be investigated. The present radiological (ATVA) marker may potentially identify inner ears at risk even before any MD-associated functional changes occur, i.e., in early childhood, when the temporal bone anatomy is matured. However, its validity for predicting MD in asymptomatic individuals with ES hypoplasia needs to be investigated.

Regarding the potential clinical use of the ATVA marker, the following points should further be considered: (i) the time interval from initial MD diagnosis to bilateral affection is highly variable among patients (4, 5, 11, 28, 29) and cannot be predicted with the ATVA marker; (ii) the measurement of an α_exit_ of <120° in the contralateral ear does not with absolute certainty exclude progression to bilateral disease, since a different MD-causing process can affect this ear (i.e., another MD endotype) (9, 11), although this remains hypothetical; and (iii) the determination of the ATVA on both sides in Gd-MRI (3D IR sequence) data requires systemic (i.v.) delivery of the Gd-contrast agent.

In conclusion, ongoing observations will determine whether the promising preliminary data hold up and whether both study cohorts remain “true to prediction,” thus ultimately validating the ATVA marker as a prognostic tool for bilateral disease progression in MD-hp patients.

## Data Availability Statement

The raw data supporting the conclusions of this article will be made available by the authors, without undue reservation.

## Ethics Statement

The studies involving human participants were reviewed and approved by Ethikkommission Zürich, KEK-ZH-Nr. 2016-01619/2019-01006. The patients/participants provided their written informed consent to participate in this study.

## Author Contributions

The study was concepted and designed by DB and AE. Acquisition and analysis of data were performed by DB, BS, JD, and AE. DB and AE drafted the manuscript. All authors critically reviewed and revised the manuscript.

## Conflict of Interest

The authors declare that the research was conducted in the absence of any commercial or financial relationships that could be construed as a potential conflict of interest.

## Abbreviations

ATVA: angular trajectory of the vestibular aqueduct
cVEMP: cervical vestibular-evoked myogenic potential
ES: endolymphatic sac
Gd-MRI: gadolinium-enhanced MRI
HRCT: high-resolution CT
MD: Meniere's disease
MD-dg: Meniere's disease associated with a degeneration of the endolymphatic sac (and a normal vestibular aqueduct)
MD-hp: Meniere's disease associated with a developmental hypoplasia of the vestibular aqueduct and endolymphatic sac.
